# Functional Development of the Octenol Response in *Aedes aegypti*

**DOI:** 10.3389/fphys.2013.00039

**Published:** 2013-03-07

**Authors:** Jonathan D. Bohbot, Nicolas F. Durand, Bryan T. Vinyard, Joseph C. Dickens

**Affiliations:** ^1^Invasive Insect Biocontrol and Behavior Laboratory, Henry A. Wallace Beltsville Agricultural Research Center, Plant Sciences Institute, United States Department of Agriculture, Agricultural Research ServiceBeltsville, MD, USA; ^2^Biometrical Counseling Service, Henry A. Wallace Beltsville Agricultural Research Center, United States Department of Agriculture, Agricultural Research ServiceBeltsville, MD, USA

**Keywords:** odorant receptor, gustatory receptor, octenol, CO_2_, olfaction, *Aedes aegypti*, development

## Abstract

Attraction of female *Aedes aegypti* mosquitoes to 1-octen-3-ol (octenol), CO_2_, lactic acid, or ammonia emitted by vertebrate hosts is not only contingent on the presence of odorants in the environment, but is also influenced by the insect’s physiological state. For anautogenous mosquito species, like *A. aegypti*, newly emerged adult females neither respond to host odors nor engage in blood-feeding; the bases for these behaviors are poorly understood. Here we investigated detection of two components of an attractant blend emitted by vertebrate hosts, octenol, and CO_2_, by female *A. aegypti* mosquitoes using electrophysiological, behavioral, and molecular approaches. An increase in sensitivity of octenol olfactory receptor neurons (ORNs) was correlated with an increase in odorant receptor gene (*Or*) expression and octenol-mediated attractive behavior from day 1 to day 6 post-emergence. While the sensitivity of octenol ORNs was maintained through day 10, behavioral responses to octenol decreased as did the ability of females to discriminate between octenol and octenol + CO_2_. Our results show differing age-related roles for the peripheral receptors for octenol and higher order neural processing in the behavior of female mosquitoes.

## Introduction

Female *Aedes aegypti* mosquitoes spread human pathogenic viruses that cause yellow fever, dengue fever, and Chikungunya. Much of the struggle against these diseases has relied on a combination of prophylactic measures such as vector control including insecticides and odor-baited traps. These systems can be improved once we better understand the relationships between the *A. aegypti* olfactory system and odorants.

**Figure 1:**
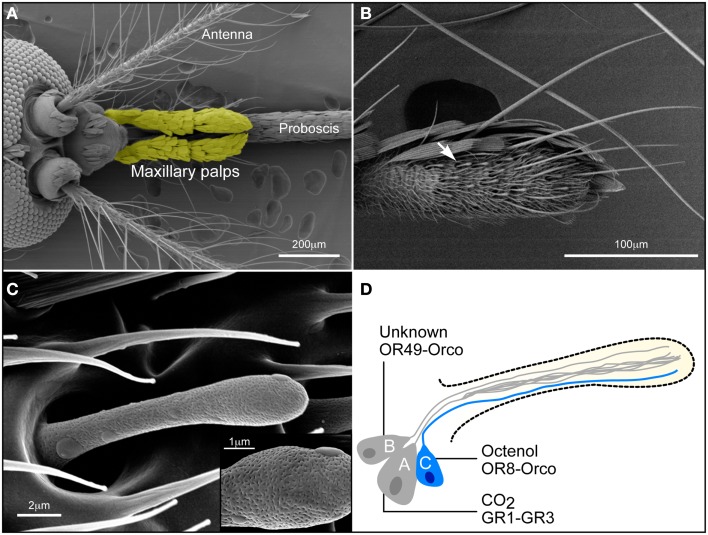
**Olfactory system of the female *Aedes aegypti* mosquito**. **(A)** The *A. aegypti* head (dorsal view) possesses three olfactory appendages: the antennae, the maxillary palps (here colored yellow using Adobe Photoshop), and the proboscis. **(B)** Basiconic sensilla (white arrow) are the only chemosensory organs on the fourth segment of the maxillary palps (ventro-lateral view). **(C)** A basiconic sensillum is a club-shaped sensory organ. Pores (see insert) allowing odorants to penetrate the sensillum are densely distributed over the surface of the hair. **(D)** Diagram outlining the conceptual neuronal content of a basiconic sensillum. Three chemosensory neurons are enclosed within the sensillum. The CO_2_ or “A” neuron expresses *AaGr1* and *AaGr3*. The “B” neuron is presumed to express *AaOr49-Orco* (unknown ligand). The *Aa*OR8-Orco complex confers octenol sensitivity to the “C” neuron (labeled in blue).

The olfactory system of insects is divided into olfactory receptor neurons (ORNs), which constitute the peripheral olfactory system, and higher brain centers which receive messages from the ORNs (Galizia and Rossler, 2010). Sensilla distributed on the antennae, maxillary palps, and proboscis of mosquitoes (Figure 1A) house ORNs that detect volatile chemicals and transduce these signals into electrical outputs for further processing within the central nervous system. Detection of specific odorants, such as CO_2_, octenol, lactic acid, and ammonia, is carried out by receptor proteins located in the dendritic membrane of ORNs; these receptors belong to the odorant receptor (OR), gustatory receptor (GR), and ionotropic receptor classes (Hansson and Stensmyr, 2011).

**Figure 2:**
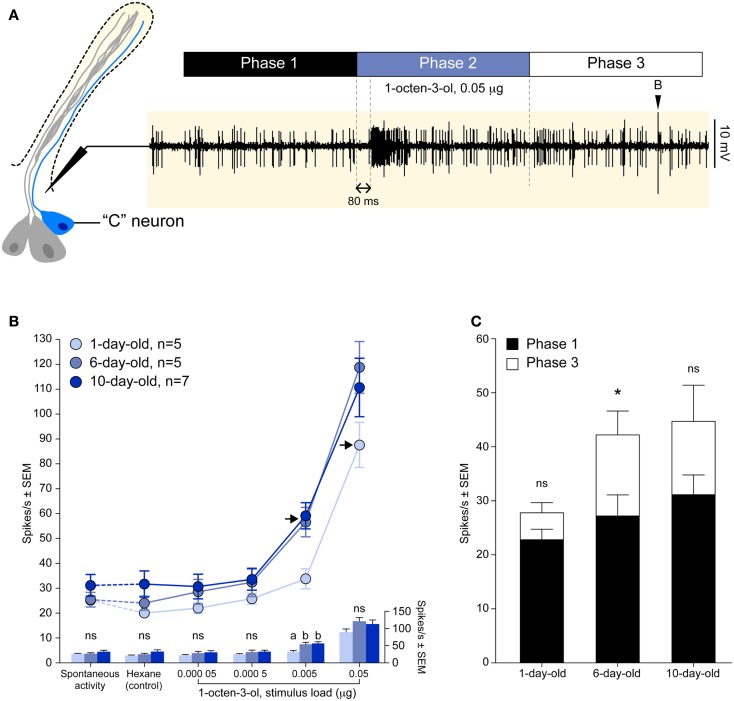
**Physiological development of the octenol receptor neuron**. **(A)** Diagram outlining the methodology used to record action potentials of the “C” neuron in response to 1 s exposure of CO_2_-free air, hexane, or octenol (phase 2). A delay of 80 ms was observed between stimulation and the response. The use of CO_2_-free air allowed for the unobstructed observation of medium (inverted black triangle) and small-sized spikes, the latter being elicited by octenol. **(B)** Dose-dependent curves of mean octenol responses from the “C” neuron of 1, 6, and 10 days old adult female mosquitoes. Points represented are mean ± SEM (*n* = 5–7). Responses to octenol were compared to hexane control using a one-way ANOVA followed by Dunnett’s post test (***P < 0.001; **P < 0.01; ns, not significant). Detection threshold for 1 day old and 6/10 days old insects was 0.05 and 0.005 μg, respectively (black arrows). The histograms below shows the comparison of the mean responses for each concentration (one-way ANOVA followed by Tukey’s multiple comparison post test, *P* < 0.05; ns, not significant). **(C)** The rate of spontaneous activity pre-stimulation (phase 1) and post-stimulation (phase 3) showed a significant change for 6 days old insects (Student *t* test, *P < 0.05; ns, not significant).

Maxillary palps (Figure 1A) of female *A. aegypti* harbor 29–35 club-shaped basiconic sensilla (McIver, 1982; Figure 1B) that house specific ORNs that detect the attractants octenol and CO_2_ (Takken and Kline, 1989; Grant and O’Connell, 1996). These multiporous basiconic sensilla (Figure 1C) are the only chemosensory organs found on the maxillary palps and are innervated by three ORNs (Figure 1D), each generating a characteristic action potential. The largest amplitude action potential from the “A” neuron responds to fluctuations of CO_2_ (Grant and O’Connell, 1996). A cognate stimulus is unknown for the “B” neuron characterized by an intermediate amplitude spike (Figure 2A). The “C” neuron, which produces the smallest amplitude spike (Figure 2A) responds to the *R*-enantiomer of octenol (Grant and Dickens, 2011).

Studies in *Anopheles gambiae* have shown that the “C” neuron co-expresses the *A. gambiae* GR 22 (*AgGr22*), *AgGr23*, and *AgGr24* genes (Jones et al., 2007; Lu et al., 2007), the orthologs of the *Drosophila melanogaster* CO_2_ receptors *Gr21a* and *Gr63a* (Jones et al., 2005; Kent et al., 2008; Robertson and Kent, 2009). Three orthologs have been identified in the maxillary palps of *A. aegypti*: *AaGr1*, *AaGr2*, and *AaGr3* (Kent et al., 2008; Robertson and Kent, 2009). While both *AaGr1* and *AaGr3* have been implicated in CO_2_ sensing, the role of *AaGr2* is unknown (Erdelyan et al., 2011). The receptor assemblage comprised of the odorant sensing subunit AaOR8 and the obligatory coreceptor AaOrco specifically recognizes (*R*)-octenol (Bohbot and Dickens, 2009) and provides enantioselectivity to the “C” cell (Grant and Dickens, 2011). While there is no direct evidence of *AaOr8* expression in the dendritic segment of the “C” cell, the *A. gambiae Or8* ortholog is present in one of the three ORNs in the basiconic sensilla and *AaOr8* is expressed in the maxillary palp (Bohbot et al., 2007).

Since only three *Ors* have been identified in the maxillary palps (Bohbot et al., 2007), the current model assumes that *AaGr1, 2, 3* are expressed in the “A” neuron while Aa*Or49-Orco* and Aa*Or8-Orco* are expressed in the “B” and “C” neurons, respectively (Figure 1D).

For holometabolous insects, like mosquitoes, the adult phenotype is acquired during the metamorphic pupal stage. The adult peripheral sensory apparatus develops from imaginal disks and the brain undergoes dramatic remodeling. These profound changes accompany the organism’s transition from an aquatic to a terrestrial life style, and ends shortly after emergence (Clements, 1999). In their first 24 h as imago, female mosquitoes do not blood-feed and their sensory capability is incomplete. For example, the physiological development of CO_2_-sensitive neurons on the palps of newly emerged female *A. aegypti* is age-dependent (Grant and O’Connell, 2007), much like the lactic-acid sensitive neuron on the antennae (Davis, 1984). It is unknown whether the physiology of the octenol-sensitive “C” neuron follows a similar developmental pattern in newly emerged and older adults.

In this study, we have focused our attention on the developmental aspects of octenol detection in adult female *A. aegypti* mosquitoes using molecular, cellular, and behavioral approaches. We show that genetic and physiological components directly involved in octenol detection are maturing 24 h post-emergence. Individual 1 day old mosquitoes show no chemotaxis toward either CO_2_ or CO_2_ + octenol while older animals are attracted by both. Our data indicate that the peripheral sensory system responsible for octenol detection is not mature at emergence, and suggest that associated behavioral responses to CO_2_ and CO_2_ + octenol are under temporal control by the peripheral and central olfactory systems.

## Materials and Methods

### Insects

Eggs of the Orlando (1952 Florida) strain of *A. aegypti* (L.) were obtained from The Center for Medical and Veterinary Entomology, USDA, ARS in Gainesville, FL, USA. Mosquitoes were reared in an environmental chamber at 27°C and 70% relative humidity with a 12:12 light-dark cycle. Larvae were fed on a fish food diet (Tetramin^®^). Unsexed pupae were hand-collected and placed into small cages (9 cm × 5.5 cm). Adults consistently emerged 2 days following pupation and were segregated according to their emergence date. Male and female adult mosquitoes were allowed to mate and were fed on a 10% sucrose solution *ad libitum*.

### RNA isolation

Five hundred and ten maxillary palps were dissected for each age group (1, 6, and 10 days old animals). Each tissue collection was placed in dry ice and mechanically ground in TRIzol^®^ (Invitrogen Life Technologies, Carlsbad, CA, USA). Total RNA was isolated following the manufacturer’s protocol and sent to the Genomic Services Lab at Hudson Alpha Institute for Biotechnology (Huntsville, Alabama). Messenger RNA isolation and cDNA synthesis were prepared using the Illumina^®^ TruSeq™ RNA Sample Preparation Kit (Illumina Inc., San Diego, CA, USA). Three libraries were sequenced and carried out on an Illumina HiSeq2000 to generate 50 bp paired-end reads.

### Post-sequencing analysis of RNA-seq data

Reference genome and annotations for *A. aegypti* (AaegL1.3) were downloaded from VectorBase^1^. Output Fastq Illumina files were mapped to the reference genome with TopHat (Trapnell et al., 2009). Resulting sequence alignment files were uploaded into the Avadis NGS software (Strand Scientific Intelligence, CA, USA), where quantification and normalization were carried out. Prior to quantification using the Deseq normalization method, the read list was filtered to remove duplicate, single end, mate filtered, mate missing, one mate flip, both mate flip, and unaligned reads. Transcript expression levels for *AaOr8* (gene accession number: AAEL012254), *AaOr49* (AAEL001303), *AaOrco* (AAEL005776), *AaGr1* (AAEL002380), *AaGr2* (AAEL002167), and *AaGr3* (AAEL010058) were reported in units of Reads Per Kilo-base per Million reads mapped (RPKMs).

### Sensory physiology

Single-cell electrophysiological recordings were carried out on the basiconic sensilla of the maxillary palp of 1, 6, and 10 days old female *A. aegypti* as previously described (Grant and Dickens, 2011). Mosquitoes were immobilized by brief chilling at −20°C and glued on their side to a mounting stage in order to position the basiconic sensilla on the maxillary palp for access by the recording electrode. Both the recording and reference tungsten electrodes were electrolytically sharpened to a tip of less than 1 μm diameter. The recording electrode was placed at the base of a basiconic sensillum while the reference electrode was inserted into the insect’s compound eye. Serial dilutions of racemic octenol (>98%, Fluka Chemical Corp., Milwaukee, WI, USA) were prepared in spectrophotometric grade hexane. Volatiles emanating from 5 μL aliquots of the serial dilutions applied to filter paper strips were carried over the preparation by compressed air (Ultra Zero Grade, >0.5 ppm CO_2_, 665 mL/min). Stimulus duration was 1 s. The analog signal was amplified and filtered (bandpass 300–1000 Hz) using a preamplifier (model P15D, Grass Instrument Corp., Quincy, MA, USA). Further amplification and signal digitization were performed by an IDAC 4 (Syntech, Kirchzarten, Germany). Data were visualized, recorded, and analyzed using AutoSpike (Syntech, Kirchzarten, Germany) software and a microcomputer. The smallest amplitude spikes were invariably elicited by octenol. Spikes were counted for three time periods: 1 s prior to stimulus onset (Phase 1), the 1 s stimulus duration (Phase 2), and 1 s following the stimulus (Phase 3; Figure 2A).

### Bioassay

**Figure 3:**
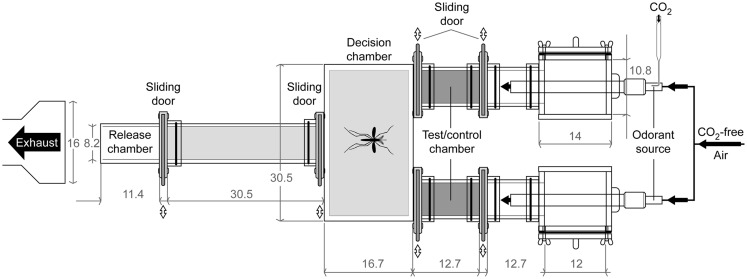
**Technical 2D-diagram of the dual-choice bioassay**. Mosquitoes trapped in the area outside the release and test/control chambers (light gray area) were counted as activated. Mosquitoes that have flown to the test/control chambers (dark gray areas) were counted as attracted. Dimensions given in cm.

Behavioral bioassays of single mosquitoes were conducted using a Plexiglas^®^ dual-choice olfactometer (custom made by Precision Plastics Inc., Beltsville, MD, USA, Figure 3) designed to measure upwind attraction, modified after Geier et al. (1999) and Bosch et al. (2000). The most upwind section of the olfactometer consisted of two 12.7 cm lengths plastic cylinders (diameter 10.2 cm) containing aluminum tubes (diameter 2 cm, length 30 cm). A laminar flow was made by passing the airflow through a spongy steel insert within the tubes. Air flowed through each of the two cylinders, through mesh screens (to prevent mosquito entry), and into the cylinders (inner diameter 10.2 cm, 12.7 cm long) corresponding to the control chamber and the test chamber (in which volatile stimuli were introduced). Each chamber contained a sliding screen door which could be closed at the conclusion of each experiment. The two chambers were attached to a rectangular box (10.8 cm H × 30.5 cm W × 17.1 cm L) opposite a single cylinder (10.2 cm inner diameter, 30.5 cm long) where mosquitoes were individually released from cages. Release cages were cylinders (10.2 cm inner diameter, 11.4 cm long) with screen covering one end and a sliding door with screen on the other end. All parts were designed to be easily removable prior to cleaning with distilled water and Alconox^®^ laboratory detergent. Experiments were conducted under red light conditions using four Safelight lamps (Kodak, USA) equipped with Roscolux medium red filters (Roscolab Ltd., London, UK) mounted 35.6 cm above the olfactometer. Intensity and wavelength of emitted light was measured with a spectrometer (USB4000, Ocean Optics, Dunedin, FL, USA).

Air supplied by a pump located outside of the bioassay room (Gast, MI, USA) was filtered with a ultra-high capacity hydrocarbon trap (Restek, PA, USA) and humidified by passing through deionized water. The airstream was maintained at 27°C and 60–73% relative humidity as measured by a hygrothermometer (EA80, Extech Instruments, Waltham, MA, USA). The flow rate was 6 L/min (30 ± 15 cm/s airspeed as measured by a hot-wire anemometer, model 441S, Kurtz, CA, USA; accuracy ±0.01 m/s).

Custom air comprised of 4.5% CO_2_ (AirGas Inc., NH, USA) was regulated by a flowmeter (model N082-03 Cole-Parmer, IL, USA) at 1.6 L/min. A stimulus controller (type CS-55, Syntech, The Netherlands) was programmed for 2 s puffs at 2 s intervals to simulate discontinuous human breath. Puffs of CO_2_ were injected into the continuous air flow through a curved Pasteur pipette inserted through a hole in the aluminum tube carrying the airstream in test chamber.

Octenol was diluted in hexane to make a 10 μg/μL solution as in the physiological studies. Ten microliters of this solution was applied on a filter paper strip (size 1 cm × 4.5 cm, Whatman No. 1) to give 100 μg of 1-octen-3-ol on the paper. The hexane solvent was allowed to evaporate under a hood for approximately 20 s at room temperature prior to placing the filter paper in the aluminum tube of the test chamber.

### Behavioral test

Non-blood-fed female mosquitoes were aspirated individually into release cages 20 min prior to the experiment. Mosquitoes were acclimated for 10 min under normal light then 10 min under red light. The test was started by slowly opening the release cage. Each mosquito was observed for 10 min for the following behaviors: activation (mosquito took flight and left the release chamber) and attraction (mosquito entered the control or test chamber). The insect was then removed from the olfactometer and odorant-free air flowed through the device to remove any contaminant for 5 min prior to the next experiment. Between each test, the control and test chambers were inverted to avoid any preference for the left or the right arm of the olfactometer. CO_2_ alone and CO_2_ + octenol were alternated and 20 individuals were tested for each treatment. Tests were conducted from 10:00 to 17:00 h during the light cycle under which the insects were reared.

### Bootstrap analysis

The observed data, 20 mosquitoes for each of the three ages × two treatment groups, although time consuming and labor intensive to bioassay, were inadequate sample sizes to produce estimates of the proportion of mosquitoes attracted with sufficient accuracy and precision to statistically detect differences of practical biological importance. Therefore, the original observed data set, was considered to be one replicate of the experiment, and was resampled to acquire bootstrap (Manly, 2007) replicates of the experiment. Estimates of the proportion of mosquitoes attracted to a stimulus were obtained by fitting an over-dispersed Binomial model using the glmer function in the lme4 package in the *R*^2^ statistical freeware environment. The over-dispersed Binomial ANOVA model was fit to the observed data, then to the observed data plus the first resampled replicate (i.e., two full replicates of the experiment). Each time another resampled replicate was added to the data set, the ANOVA model was fit again; allowing identification of a necessary and sufficient amount of replication for statistical detection of biologically and practically important differences (when they exist) in proportion of mosquitoes attracted.

## Results

### Electrophysiological response of octenol-sensitive neurons

We recorded the electrical activity of the “C” neuron (smallest amplitude spike) using the single-cell recording technique in 1, 6, and 10 days old adult female *A. aegypti* mosquitoes. The activity of the “C” neuron was divided into three 1 s periods: before stimulation (phase 1), during stimulation (phase 2), and after stimulation (phase 3; Figure 2A).

The firing rate of the “C” neuron elicited by hexane and increasing stimulus loads of racemic 1-octen-3-ol were analyzed during phase 2 (Figure 2B). The firing rate evoked by hexane did not differ from the rate of the spontaneous activity, and the three age groups did not differ in their rate of spontaneous activity (one-way ANOVA, *P* = 0.4044; Figure 2B). The detection threshold was determined by comparing responses elicited by serial dilutions of octenol to the hexane control. Responses to 0.05 μg octenol was the lowest dose statistically different from hexane in 1 day old animals. The detection threshold for 6 and 10 days old animal was 10 times lower at 0.005 μg octenol (Figure 2B). While the detection threshold was clearly established for the three age groups, we did not detect a significant difference with the highest stimulus load (Figure 2B).

The physiological recovery of the “C” neuron was assessed by comparing the rate of the spontaneous activity 1 s before (phase 1) and 1 s after (phase 3) stimulation (Figure 2A). Spontaneous activity showed a statistically different (Student *t* test, *P* < 0.05) recovery rate in 6 days old animals at the highest concentration (0.05 μg; Figure 2C). For all other concentrations and for all other age groups the rate of spontaneous activity always recovered during phase 3 (data not shown).

### Development of behavior: Activation and attraction

**Figure 4:**
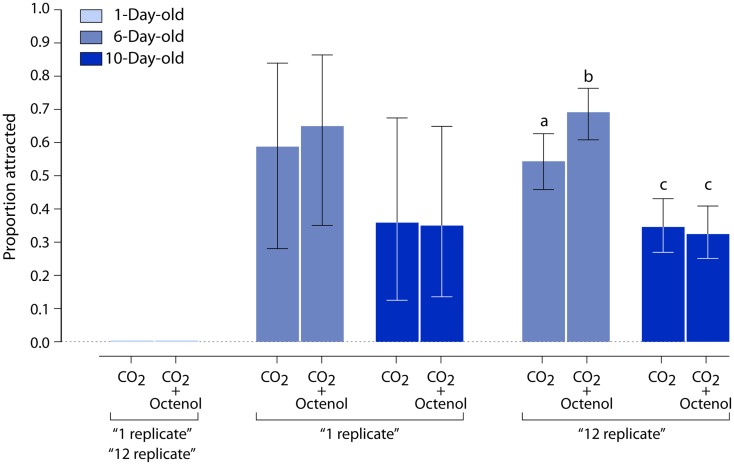
**Influence of age on behavioral responses to CO_2_ and octenol**. Newly emerged female mosquitoes (1 day old) are neither activated by CO_2_ nor attracted to CO_2_ + octenol. Six day old individuals show significant attraction to both CO_2_ and CO_2_ + octenol. Ten day old animals exhibit lower CO_2_ activation and no attraction to CO_2_ + octenol. Vertical bars represent 95% confidence intervals.

Except during mating, when adult males form occasional swarms to attract females (Goeldi, 1905; Hartberg, 1971; Cabrera and Jaffe, 2007), mosquitoes have a solitary lifestyle. Thus, individual females were tested in our dual-choice bioassay (Figure 3). Carbon dioxide, a known activator of upwind flight, was used alone or in the presence of octenol. We looked at female mosquitoes distributed in three age groups for a total of six experiments. One day old animals were not attracted to either CO_2_ alone or CO_2_ + octenol (Figure 4). While CO_2_ alone and CO_2_ + octenol elicited strong attraction from 6 and 10 days old animals, we could not detect any difference between the two stimuli and the two age groups.

Simple ANOVA analysis did not identify a significant difference between the two stimuli at either of the older age groups. Since the nature of the bioassay of individual mosquitoes was time consuming and labor intensive, additional replicate observations were impractical. Therefore, we adopted a simulation-based approach to generate bootstrap samples. The observed data, one replicate of the experiment, was resampled to acquire bootstrap (Manly, 2007) replicates of the experiment, to determine potential outcomes of additional sampling. The “six replicate” result, the observed data plus five resampled replicates, was the smallest number of replicates to show a reduced attraction to both treatments in 10 days old insects (*P* < 0.001; data not shown). The “12 replicate” bootstrap result indicated that CO_2_ + octenol was more attractive than CO_2_ alone in 6 days old animals (*P* < 0.01; Figure 4).

### Profiling chemosensory gene expression

**Figure 5:**
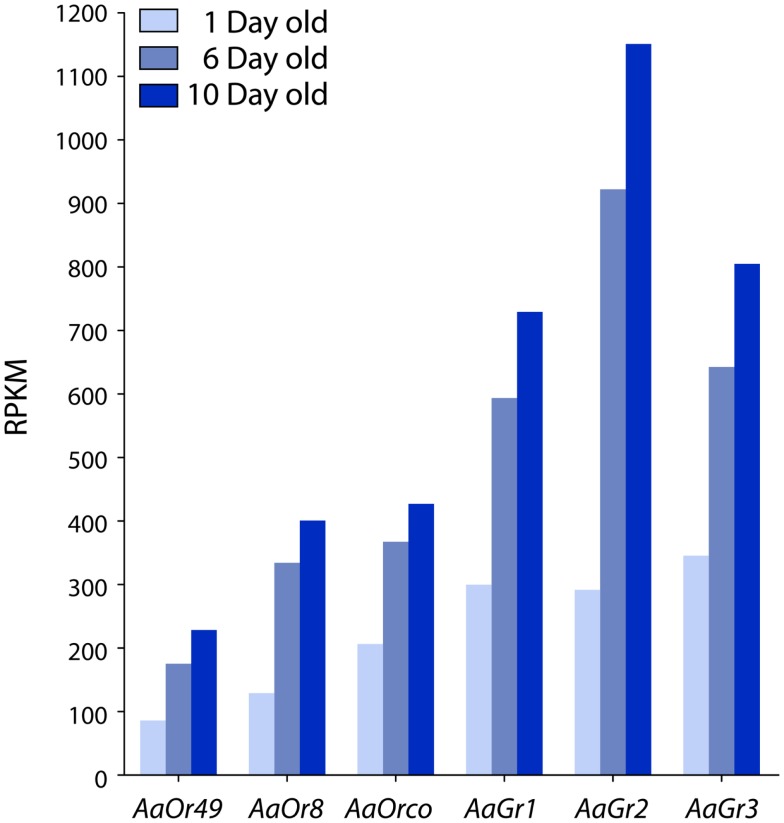
**Temporal expression of *AaOr* and *AaGr* genes in the maxillary palps of female *Aedes aegypti* defined by RNA-seq**. All genes were highly expressed throughout adulthood. The largest increase in expression occurs between 1 and 6 days old animals, and is followed by a plateau in 10 days old mosquitoes.

High-throughput sequencing (RNA-seq) was used to identify and measure the abundance of expressed genes in the maxillary palps of 1, 6, and 10 days old female mosquitoes. Among the thousands of genes present in each sample, we chose to focus on chemosensory receptor genes known to be expressed in the palps including *AaOr8*, *AaOr49*, *AaOrco*, *AaGr1*, *AaGr2*, and *AaGr3*. *AaOr8-Orco*, *AaGr1*, and *AaGr3* are presumably expressed in the basiconic sensilla based on their function in octenol (Bohbot and Dickens, 2009) and CO_2_ reception (Erdelyan et al., 2011), respectively. All six genes exhibited the same general trend: robust expression at emergence with increased expression occurring between 1 and 6 days old animals before some leveling off in 10 days old insects (Figure 5).

## Discussion

Single-cell recordings and molecular expression data indicate that at emergence, octenol-sensitive neurons are physiologically competent, but have not reached maturity. We have confirmed that *AaOr8* and *AaOrco* expression levels are significant in newly emerged mosquitoes (Bohbot et al., 2007) and continue to increase after adult emergence. Absolute RPKM (estimation of gene expression) values were consistent with previously described levels of transcripts in the antennae of *A. gambiae* (Pitts et al., 2011), confirming that these genes are highly expressed in the maxillary palps of *A. aegypti*. Although we did not report evidence that the encoded proteins are present in the dendritic processes of the octenol ORNs, based on the observed physiological responses, we speculate that protein translation reflects RNA expression levels. The same observation can be made for *AaGr1* and *AaGr3* (CO_2_ receptor), as well as for *AaGr2* and *AaOr49*. A previous study showed that *AaGr1, 2, 3* expression begins during the pupal stage and continues into adulthood (Erdelyan et al., 2011), which is consistent with our gene expression analysis.

Maturation of neuronal responsiveness to octenol resembles the developmental pattern of the CO_2_ response (Grant and O’Connell, 2007) in female *A. aegypti*. This similarity may be a result of common morphological and hormonal factors in sense organ development (Clements, 1999) as both neurons occupy the same basiconic sensilla on the palps (McIver, 1972). According to Clements (1999), the first 24 h following emergence is a vulnerable period for female *A. aegypti* whose various parts of the body, including the mouthparts, are undergoing hardening or sclerotization. Coincidently, these young insects exhibit reduced physiological competency, do not show any behavioral attraction to a human hand, and do not blood-feed. Davis (1984) showed that the sensitivity of lactic-acid sensitive neurons develops with age and is also dependent on the blood-feeding status of female *A. aegypti*. Juvenile hormone released by the corpora allata has been implicated in regulating biting behavior in newly emerged *Culex* mosquitoes (Meola and Petralia, 1980) and might play a role in *A. aegypti* (Klowden and Blackmer, 1987). This would indicate that a neural or humoral mechanism is preventing newly emerged female mosquitoes from feeding.

Could a similar mechanism govern cuticle hardening and neural maturation? Both the molecular and physiological data reported here indicate that the peripheral olfactory system has reached sufficient maturity to provide sensory input for eliciting behavior. However, behavioral responses in the form of attraction to CO_2_ or CO_2_ + octenol were absent, which indicates a deficit of responsive central neural pathways or a humoral control mechanism of these pathways. Presynaptic and postsynaptic modulation of olfactory processing (Wang, 2011) have been reported in multiple insect species and involve biogenic amines (Kuppers and Thürm, 1975; Linn and Roelofs, 1986; Pophof, 2000, 2002; Grosmaitre et al., 2001; Spivak et al., 2003; Zhukovskaya and Kapitsky, 2006; Flecke and Stengl, 2009; Vergoz et al., 2009; McQuillan et al., 2012), dopamine (Andersen et al., 2006), tachykinins (Winther et al., 2006; Ignell et al., 2009), and short neuropeptide F (Root et al., 2011), as well as insulin (Root et al., 2011).

The activation and attraction elicited by CO_2_ and CO_2_ + octenol in 6 days old animals, and the following reduction of the same behaviors associated with 10 days old animals, despite full peripheral olfactory competency, suggest central nervous system modulation by some humoral factor. Another possibility is that senescence, defined by a post-maturation decline in cell integrity and function, may be the cause of reduced activation and attraction in older insects (Seabrook et al., 1979, 1987; Fescemyer and Hanson, 1990).

In conclusion, our results, taken together with previous investigations, show that the physiological development of octenol, lactic acid, and CO_2_-sensitive receptor neurons are synchronous and age-dependent in adult mosquitoes. Our data indicate concerted changes in chemosensory gene expression that correlate with increased olfactory sensitivity between 1 and 6 days old insects. We speculate that for older animals, age leads to an inevitable decline in sensory function as shown for other insects (Seabrook et al., 1979; Dickens and Moorman, 1990). As octenol, CO_2_, lactic acid, and other chemicals are vital chemical cues for the ecological success of mosquitoes (Takken and Knols, 1999), it is important to understand which of these sensory modalities may provide the most stable targets for chemical disruption.

## Conflict of Interest Statement

The authors declare that the research was conducted in the absence of any commercial or financial relationships that could be construed as a potential conflict of interest.

## Footnotes

^1^http://aaegypti.vectorbase.org/GetData/Downloads/

^2^http://www.r-project.org
